# Using Virtual Reality to Improve Health Care Providers’ Cultural Self-Efficacy and Diabetes Attitudes: Pilot Questionnaire Study

**DOI:** 10.2196/23708

**Published:** 2021-01-27

**Authors:** Elizabeth Ann Beverly, Carrie Love, Matthew Love, Eric Williams, John Bowditch

**Affiliations:** 1 Department of Primary Care Heritage College of Osteopathic Medicine Ohio University Athens, OH United States; 2 Game Research and Immersive Design Lab J Warren McClure School of Emerging Communication Technologies Ohio University Athens, OH United States

**Keywords:** virtual reality, diabetes attitudes, cultural self-efficacy, health care providers, VR, diabetes, training

## Abstract

**Background:**

In southeastern Appalachian Ohio, the prevalence of diabetes is 19.9%, nearly double that of the national average of 10.5%. Here, people with diabetes are more likely to have a delayed diagnosis, limited access to health care, and lower health literacy. Despite the high rates of diabetes in the region, the availability of endocrinologists and certified diabetes care and education specialists is limited. Therefore, innovative strategies to address the growing diabetes care demands are needed. One approach is to train the primary care workforce in new and emerging therapies for type 2 diabetes to meet the increasing demands and complexity of diabetes care.

**Objective:**

The aim of this study was to assess the effectiveness of a virtual reality training program designed to improve cultural self-efficacy and diabetes attitudes.

**Methods:**

Health care providers and administrators were recruited from large health care systems, private practices, university-owned hospitals or clinics, Federally Qualified Health Centers, local health departments, and AmeriCorps. Providers and administrators participated in a 3-hour virtual reality training program consisting of 360-degree videos produced in a professional, cinematic manner; this technique is called virtual reality cinema (cine-VR). Questionnaires measuring cultural self-efficacy, diabetes attitudes, and presence in cine-VR were administered to providers and administrators before and after the program.

**Results:**

A total of 69 participants completed the study. The mean age of the sample was 42.2 years (SD 13.7), 86% (59/69) identified as female, 83% (57/69) identified as White, 86% (59/69) identified as providers, and 25% (17/69) identified as nurses. Following the training program, we observed positive improvements in all three of the cultural self-efficacy subscales: *Cognitive* (mean change –1.29; *t_65_*=–9.309; *P*<.001), *Practical* (mean change –1.85; *t_65_*=–9.319; *P*<.001), and *Affective* (mean change –0.75; *t_65_*=–7.067; *P*<.001). We observed the largest magnitude of change with the subscale, with a Cohen *d* of 1.16 indicating a very large effect. In addition, we observed positive improvements in all five of the diabetes attitude subscales: Need for special training (mean change –0.21; *t_67_*=–6.154; *P*<.001), Seriousness of type 2 diabetes (mean change –0.34; *t_67_*=–8.114; *P*<.001), Value of tight glucose control (mean change –0.13; *t_67_*=–3.029; *P*=.001), Psychosocial impact of diabetes (mean change –0.33; *t_67_*=–6.610; *P*<.001), and Attitude toward patient autonomy (mean change –0.17; *t_67_*=–3.889; *P*<.001). We observed the largest magnitude of change with the Psychosocial impact of diabetes subscale, with a Cohen *d* of 0.87 indicating a large effect. We observed only one significant correlation between presence in cine-VR (ie, *Interface Quality*) and a positive change score (ie, *Affective* self-efficacy) (*r*=.285; *P*=.03).

**Conclusions:**

Our findings support the notion that cine-VR education is an innovative approach to improve cultural self-efficacy and diabetes attitudes among health care providers and administrators. The long-term impact of cine-VR education on cultural self-efficacy and diabetes attitudes needs to be determined.

## Introduction

Appalachia is a 205,000-square-mile region that encompasses 420 counties in 13 US states from Mississippi to New York. Ohio’s Appalachian region encompasses 32 counties [1], of which 16 are designated as economically *at risk* or *distressed* [2]. Here, 17.2% of the population live below the poverty line as compared to 14.4% for the rest of the state [3], and the counties with the highest poverty rates, ranging from 22.5% to 30.2%, are Appalachian [3]. People who live in Appalachian Ohio are more likely to be unemployed, have lower educational achievement, and limited access to transportation [4]. These social determinants of health contribute to the health disparities observed among people living in this region [5].

One health disparity disproportionately affecting people in Appalachian Ohio is diabetes [5]. An alarming 19.9% of adults in southeastern Ohio have diabetes [6], which is nearly double the national average of 10.5% [7]. In this region, people are more likely to have a delayed diabetes diagnosis, limited access to health care, lower health literacy, and lower empowerment [8,9]. For these reasons, people here are more likely to have macrovascular and microvascular complications, lower limb amputations, and depression [9-11]. Despite the high rates of diabetes in the region, the availability of endocrinologists and certified diabetes care and education specialists in Appalachian Ohio is limited [12]. Therefore, innovative strategies to address the growing diabetes care demands are needed.

One approach is to train the primary care workforce in new and emerging therapies for type 2 diabetes to meet the increasing demands and complexity of diabetes care. Primary care providers deliver more than 90% of the clinical care to people with type 2 diabetes in the United States [13]. This is even more pertinent in rural America where family physicians comprise a greater proportion of the workforce and provide comprehensive and irreplaceable care to the community [14]. Therefore, tailored continuing education for rural primary care providers and their staff is critical. Continuing education should address standards of medical care for diabetes as well as cultural competency and attitudes toward diabetes. Studies show that health care providers’ attitudes toward diabetes influence their approach to care (eg, paternalistic vs patient-centered care) and how they interact with people with diabetes [15-18]. Furthermore, continuing education that recognizes the unique cultural contributions of regions like Appalachian Ohio is necessary to improve providers’ ability to care for people from different backgrounds [19,20]. People from Appalachia share common language, behaviors, dietary habits, and value systems. Health care providers who understand their patients’ cultural backgrounds are more likely to observe improvements in diabetes outcomes and patient satisfaction [21,22]. Thus, tailoring continuing education to address diabetes attitudes and Appalachian culture is critical to improve the quality of care to an ever-increasing number of people with diabetes in Appalachian Ohio.

Virtual reality cinema (cine-VR) is an innovative educational technique that has the potential to transform the delivery and content of continuing medical education. Cine-VR is dynamic, accessible, and adaptable to providers’ needs and preferences [23]. Cine-VR gives providers access to life-like medical encounters without risk or harm to the patient. Further, cine-VR offers providers a glimpse into the lives of patients and culture of the region. These qualities are invaluable to geographically and culturally distinct regions like Appalachian Ohio.

For this study, we developed a 3-hour cine-VR training program designed to educate providers and administrators about diabetes, social determinants of health, and Appalachian culture. The aim of the study was to assess the effectiveness of cine-VR training in improving health care providers’ and administrators’ cultural sensitivity and diabetes attitudes. We hypothesized that cine-VR training would improve cultural self-efficacy and diabetes attitudes.

The following are our hypotheses:

1. Levels of cultural self-efficacy will increase after the 3-hour cine-VR training program.
2. Diabetes attitudes will improve after the 3-hour cine-VR training program.
3. Positive changes in cultural self-efficacy will be associated with increased presence in the cine-VR scenarios.
4. Positive changes in diabetes attitudes will be associated with increased presence in the cine-VR scenarios.

## Methods

### Overview

The purpose of this pilot study was to call attention to social determinants of health and Appalachian culture and to delineate their relationship to diabetes via 360-degree cine-VR simulations. Specifically, we administered questionnaires to providers and administrators before and after a cine-VR training program in order to (1) assess changes in cultural self-efficacy pre- and posttraining, (2) assess changes in diabetes attitudes pre- and posttraining, and (3) examine the relationship between changes in cultural self-efficacy and diabetes attitudes and presence in cine-VR. The Ohio University Office of Research Compliance approved the protocol (Institutional Review Board No. 19-X-99) and all recruitment procedures and materials.

### Recruitment

Providers and administrators were recruited from large health care systems, private practices, university-owned hospitals or clinics, Federally Qualified Health Centers, local health departments, and AmeriCorps. In Appalachian Ohio, the majority of providers practiced at large health care systems and Federally Qualified Health Centers. Specifically, participants were recruited via emails from the Ohio University Diabetes Institute listserv and Area Health Education Center listserv, advertisements in social media, flyers in the community, and brief announcements at educational events. Participants included physicians, nurse practitioners, registered nurses, pharmacists, dietitians, certified diabetes educators, physical therapists, dentists, community health workers, and health care administrators and staff (eg, health department employees, free clinic directors, and AmeriCorps service members). The majority of providers specialized in primary care. Health care administrators were recruited given their role in health care–related decisions and their impact on quality of care. Additionally, administrators play a significant role in the assimilation of evidence-based management and training, and cine-VR has the potential to be an evidence-based educational training model.

### Power Analysis

We conducted an a priori power analysis using Statulator [24], an online statistical calculator, which determined that a total sample size of 34 participants was estimated to achieve 80% power at a 5% significance level (*P*<.05) and to detect an effect size of 0.30.

### Cinematic 360-Degree Virtual Reality Simulations

We hosted nine 3-hour training programs in Athens, Ohio. These training programs utilized 360-degree, virtual reality, professionally produced video in a cinematic manner to educate providers and administrators about diabetes, social determinants of health, and Appalachian culture. In the *Using Virtual Reality to Visualize Diabetes in Appalachia* program, participants watched 10 cine-VR simulations and two traditional films and observed interactions among the main character and her primary care physician, pharmacist, family, and community [25]. The main character in the simulations is Lula Mae, a 72-year-old woman with type 2 diabetes living in Appalachian Ohio. She is a widow; her husband died 27 years ago from a heart attack. She has three adult children and seven grandchildren. She cares full time for her adult son who suffered a traumatic brain injury from serving in the US Army. Lula Mae and her adult son live in an old house originally belonging to her grandparents. Her two adult daughters and grandchildren live on the same family land in their separate homes. Lula Mae is a source of care and support for her entire family, from her own children to her grandchildren. In doing so, her own health care needs come second to the daily needs of the people she loves. Despite Lula Mae’s struggles, we learn about the strengths of Appalachian culture and the resiliency one person can have if providers invest the time to connect with her one-on-one.

### Training Program Curriculum

The Ohio University team developed a detailed curriculum taught synchronously with the cine-VR simulations. The curriculum included 12 modules that addressed the following content: (1) diabetes burnout, (2) food insecurity, (3) strengths of Appalachian culture, (4) rural transportation barriers, (5) elements of an effective patient-provider relationship, (6) diabetes and psychosocial issues, (7) high cost of diabetes medications, (8) gender roles in Appalachia, (9) cultural values in Appalachia, (10) diabetes complications, (11) diabetes comorbidities, and (12) patient-provider communication. An experienced behavioral diabetes researcher (EB) trained in interactive lecturing delivered all nine training sessions. The participants were encouraged to interact with each other and the lecturer. The lecturer incorporated straightforward and rhetorical questions to engage the participants. The simulations and curriculum were designed to increase cultural self-efficacy, improve diabetes attitudes, and increase presence in cine-VR. We provided 3.0 continuing medical education or continuing education credits for health care providers at no cost. Integrity of the education was ensured via a written curriculum, preapproved educational materials, and investigator observation of the training sessions.

### Virtual Reality Technology

Working with the Ohio University’s Game Research and Immersive Design Lab, we leveraged a coalition of experts from Ohio University’s Diabetes Institute and the medical school, school of nursing, social work program, nutrition program, communication sciences and disorders program, school of film, theater program, and visual communication school. The interdisciplinary team consisted of one physician, three nurses, one social worker, one clinical psychologist, one audiologist, one registered dietitian, one health behaviorist, five filmmakers, four scriptwriters, and two website developers. This collaboration allowed us to create educational content that was not only medically accurate but emotionally powerful and visually stunning. Each series began with a traditionally shot short film to set the stage between Lula Mae and her relationship with a provider. This was followed by three cine-VR simulations that opened narrative windows into her daily life, her world, and her struggles. The fifth and sixth simulations of each series were *guided simulations*, a cine-VR face-to-face conversation with Lula Mae’s provider and Lula Mae herself. This six-video pattern was repeated twice, once covering Lula Mae’s relationship with her primary care provider and once covering her relationship with her local pharmacist.

The cine-VR simulations narratively demonstrated how Lula Mae’s social determinants of health and environment shaped her behaviors. Capturing those moments with camera systems that allow the audience to see a full 360-degree sphere created opportunities to present information in ways not possible with traditional filming methods. For example, when inside Lula Mae’s home, we saw the disorganization and chaos that resulted from a lack of social support. When the family car was stranded on the side of a remote road, we saw the transportation barriers and isolation that families face in rural areas without public transportation. As a result of the 360-degree filming techniques employed, the team was able to present much more information about Lula Mae’s life and the factors affecting her diabetes.

The simulations were screened in an Oculus Go (Facebook Technologies) head-mounted display so that participants could turn their head and body in any direction and gather relevant information, much as if they were present in the actual location. Observant participants could notice subtle details, such as her surroundings, the condition of her home, or other activities co-occurring in the space. With traditionally shot films, this information would be presented in a close-up or with camera movement to call a viewer’s attention to relevant information, resulting in a more passive and guided viewing experience. Presenting the content in cine-VR creates an active viewing experience, with the viewer choosing what they want to watch and pay attention to, which increases immersion and encourages intellectual and emotional engagement. Viewers feel a sense of accomplishment as they notice subtle details planted by the filmmaking team, heightening the experience.

The fifth and sixth simulations of each series were what we called *guided simulations*, a prerecorded, cine-VR face-to-face conversation with Lula Mae’s provider and Lula Mae herself. Screened in a headset, these normally awkward, high-stakes conversations give the participants a chance to practice without the pressures of being watched or failing. Participants are encouraged to speak predetermined dialogue to a character in the headset and hear them respond. All of the cine-VR simulations were initiated simultaneously from a central computer, urging everyone in the room to say the same words at the same time, thereby reducing the potential for users to feel awkward about speaking aloud in public.

### Measures

In addition to sociodemographic factors (ie, age, sex, race or ethnicity, occupation, years in practice, health care sector, percentage of Medicaid patients, and type of Medicaid patients), participants completed the following measures.

#### Transcultural Self-Efficacy Tool–Multidisciplinary Healthcare Provider

The Transcultural Self-Efficacy Tool–Multidisciplinary Healthcare Provider (TSET-MHP) is an 83-item scale that assesses changes in self-efficacy for cultural knowledge, cultural practical skills, and cultural awareness [26]. This scale yields three subscales: (1) Cognitive, (2) Practical, and (3) Affective [27]. All three subscales are rated on a 10-point scale, ranging from 1 (not confident) to 10 (totally confident). The *Cognitive* subscale asks participants to rate their level of confidence in their knowledge of the ways cultural factors influence health care for people belonging to different cultural backgrounds. The *Practical* subscale asks participants to rate their level of confidence in interviewing people of different cultural backgrounds to learn about their values, beliefs, and social determinants of health. Lastly, the *Affective* subscale asks participants to rate their level of confidence in acceptance of similarities and differences among cultural groups. These subscales demonstrate excellent internal consistency (Cronbach α ranging from .92 to .98) [27].

#### Diabetes Attitude Scale-3

The Diabetes Attitude Scale-3 (DAS-3) [17] is a 33-item scale that measures diabetes-related attitudes with five discrete subscales: (1) Need for special training (Cronbach α=.67), (2) Seriousness of type 2 diabetes (Cronbach α=.80), (3) Value of tight glucose control (Cronbach α=.72), (4) Psychosocial impact of diabetes (Cronbach α=.65), and (5) Attitude toward patient autonomy (Cronbach α=.76). Health care professionals are asked to rate their level of agreement on a 5-point Likert scale, ranging from 1 (strongly disagree) to 5 (strongly agree). The scale demonstrates good internal consistency and high content validity [17].

#### Presence Questionnaire

The 32-item Presence Questionnaire [28] measures the subjective experience of being in a virtual environment when a person is physically situated in another. Items are rated on a 7-point scale, ranging from 1 (not at all) to 4 (somewhat) to 7 (completely). We used a subset of 15 questions from the Witmer-Singer questionnaire and removed 17 questions that measured haptic (ie, the use of technology that simulates touch) factors because the cine-VR simulations did not involve interaction with the simulated environment. For example, we removed questions that asked participants about their ability to touch objects in the virtual environment or move around in the virtual environment (eg, “How closely were you able to examine objects?” or “How compelling was your sense of moving around inside the virtual environment?”) This revised questionnaire had four subscales: (1) Involvement (Cronbach α=.83), (2) Sensory Fidelity (Cronbach α=.75), (3) Adaptation and Immersion (Cronbach α=.46), and (4) Interface Quality (Cronbach α=.53). In addition, the research team added three questions to assess presence in the virtual environment; we labeled this fifth subscale Presence (Cronbach α=.78). We calculated our own internal consistency for each subscale using a reliability analysis. The revised 18-item questionnaire demonstrated internal consistency ranging from poor to very good.

### Data Collection

At the training program, participants received a packet that included two copies of the informed consent form, a preassessment packet, and a postassessment packet. The principal investigator read the informed consent form to all attendees of the training program. Individuals interested in participating signed the informed consent form and placed it in the packet. The informed consent form emphasized the voluntary nature of participation and reminded participants that the study was not related to their participation in the overall training program. Participants completed a brief demographic form and the two preassessment questionnaires via pen and paper; this session lasted approximately 15 minutes. All questionnaires were prelabeled with an identification number prior to the start of the study. At the completion of the training program, participants completed three postassessment questionnaires via pen and paper; this session lasted approximately 15 minutes. Participants with questions about the study were directed to email or call the principal investigator (EB).

### Statistical Analysis

We assessed demographic factors using descriptive statistics and presented them as means and standard deviations or sample sizes and percentages. Chi-square tests, Fisher exact tests, independent-samples *t* tests, and one-way analyses of variance were conducted to examine differences by age, gender, race, provider status, or percentage of Medicaid (ie, limited income and resources) patients. We performed paired *t* tests to examine changes in TSET-MHP subscale scores and DAS-3 subscale scores before and after the cine-VR training program to assess changes in cultural self-efficacy and diabetes attitudes. In addition, we determined effect sizes using Cohen *d* by calculating the mean difference between the pre- and postassessment responses divided by the pooled standard deviation. Finally, we calculated mean change scores for TSET-MHP subscales and DAS-3 subscales. Then, we conducted Pearson correlations with the mean change scores for each subscale and the mean subscale scores of the Presence Questionnaire. We defined statistical significance as a *P* value less than .05 and conducted analyses in SPSS Statistics for Windows, version 26.0 (IBM Corp).

## Results

### Overview

A total of 76 individuals consented to participate in the study; however, 7 participants did not complete postsurveys. The final sample included 69 participants out of 76 (91% completion rate). The mean age of participants was 42.2 years (SD 13.7), 86% (59/69) identified as female, 83% (57/69) identified as White, 25% (17/69) were nurses, and 86% (59/69) were health care providers (see Table 1). Among health care providers, 72% (36/50) served more than 30% of patients with limited income and resources (ie, Medicaid) in their practice. The majority of providers cared for adult Medicaid patients (44/47, 94%), followed by 77% (30/39) who cared for older adults with Medicaid, and 69% (24/35) who cared for children with Medicaid.

**Table 1:** Participant demographic characteristics.

Characteristic		Participants (N=69)
Age (years), mean (SD)		42.2 (13.7)
**Gender, n (%)**		
	Female	59 (86)
	Male	10 (14)
**Race, n (%)**		
	American Indian or Alaska Native	2 (3)
	Asian Indian	1 (1)
	Black	4 (6)
	Chinese	1 (1)
	Hispanic or Latinx	2 (3)
	Other Asian	2 (3)
	White (non-Hispanic)	57 (83)
**Occupation, n (%)**		
	Community health worker	16 (23)
	Dentist	1 (1)
	Dietitian	3 (4)
	Exercise physiologist	2 (3)
	Health care administrator or staff	10 (14)
	Nurse	17 (25)
	Physician	12 (17)
	Nurse practitioner	3 (4)
	Pharmacist	4 (6)
	Physical therapist	1 (1)
**Years in health care, n (%)**		
	<1	7 (10)
	1-5	15 (22)
	6-10	6 (9)
	11-15	3 (4)
	16-20	5 (7)
	21-25	14 (20)
	26-30	4 (6)
	≥31	5 (7)
	Not applicable	10 (14)
**Health care sector, n (%)**		
	Health care system–affiliated clinic	15 (22)
	Hospital	6 (9)
	Private practice	2 (3)
	Federally Qualified Health Center	4 (6)
	Other	42 (61)
**Percentage of Medicaid patients served (n=50^a^), n (%)**		
	≤30%	9 (18)
	>30%	36 (72)
	My practice does not see Medicaid patients	5 (10)
**Age group of Medicaid patients, n (%)**		
	Children (n=35 providers)	24 (69)
	Adults (n=47 providers)	44 (94)
	Older adults (n=39 providers)	30 (77)

^a^There were 9 values missing for percentage of Medicaid patients served among the 59 providers.

### Cultural Self-Efficacy

Mean subscale scores for the TSET-MHP are presented in Table 2. Pretraining mean scores showed that the participants had the most confidence in their *Affective* cultural self-efficacy (mean 8.09, SD 1.19). Prior to the training, cultural self-efficacy scores did not differ by age, gender, race, provider status, or percent of Medicaid patients.

As hypothesized, we observed positive improvements in all three of the cultural self-efficacy subscales (see Table 2): *Cognitive* (mean change –1.29; t_65_=–9.309; *P*<.001), *Practical* (mean change –1.85; t_65_=–9.319; *P*<.001), and *Affective* (mean change –0.75; t_65_=–7.067; *P*<.001). We observed the largest magnitude of change with the *Practical* subscale, with a Cohen *d* of 1.16 indicating a very large effect. Following the training program, the cultural self-efficacy subscale scores did not differ by age, gender, race, provider status, or percent of Medicaid patients, except for postassessment *Cognitive* scores. Participants who self-identified as non-White reported greater increases than White participants in postassessment *Cognitive* subscale scores (mean difference –0.8447; t_65_=–2.021; *P*=.047).

**Table 2:** Mean differences between Transcultural Self-Efficacy Tool–Multidisciplinary Healthcare Provider (TSET-MHP) subscale scores before and after the training program.

TSET-MHP subscale	Presurvey score^a^, mean (SD)	Postsurvey score^a^, mean (SD)	*P* value	Cohen *d*
Cognitive (n=66)	6.77 (1.63)	8.06 (1.30)	<.001	0.87
Practical (n=66)	6.15 (1.78)	8.00 (1.38)	<.001	1.16
Affective (n=67)	8.09 (1.19)	8.82 (1.05)	<.001	0.66

^a^Items are rated on a 10-point scale, ranging from 1 (not confident) to 10 (totally confident).

### Diabetes Attitudes

Mean scores for the five DAS-3 subscales are presented in Table 3. Pretraining mean scores showed that participants generally agreed with the *Need for special training* (mean 4.59, SD 0.38), the *Seriousness of type 2 diabetes* (mean 4.23, SD 0.49), the *Value of tight glucose control* (mean 4.10, SD 0.40), the *Psychosocial impact of diabetes* (mean 4.43, SD 0.43), and the *Attitude toward patient autonomy* (mean 4.09, SD 0.46). No differences were observed in diabetes attitudes based on age, gender, race, provider status, or percent of Medicaid patients pretraining.

As hypothesized, we observed positive improvements in all five of the diabetes attitude subscales (see Table 3): *Need for special training* (mean change –0.21; t_67_=–6.154; *P*<.001), *Seriousness of type 2 diabetes* (mean change –0.34; t_67_=–8.114; *P*<.001), *Value of tight glucose control* (mean change –0.13; t_67_=–3.029; *P*=.001), *Psychosocial impact of diabetes* (mean change –0.33; t_67_=–6.610; *P*<.001), and *Attitude toward patient autonomy* (mean change –0.17; t_67_=–3.889; *P*<.001). We observed the largest magnitude of change with the *Psychosocial impact of diabetes* subscale, with a Cohen *d* of 0.87 indicating a large effect. Similar to the pretraining assessment, diabetes attitudes did not differ based on age, gender, race, provider status, or percent of Medicaid patients posttraining.

**Table 3:** Mean differences between Diabetes Attitude Scale-3 (DAS-3) subscale scores before and after the training program (n=68).

DAS-3 subscale	Presurvey score^a^, mean (SD)	Postsurvey score^a^, mean (SD)	*P* value	Cohen *d*
Need for special training	4.59 (0.38)	4.81 (0.27)	<.001	0.65
Seriousness of type 2 diabetes	4.23 (0.49)	4.57 (0.39)	<.001	0.78
Value of tight glucose control	4.10 (0.40)	4.24 (0.43)	.001	0.32
Psychosocial impact of diabetes	4.43 (0.43)	4.75 (0.31)	<.001	0.87
Attitude toward patient autonomy	4.09 (0.46)	4.26 (0.48)	<.001	0.38

^a^Items are rated on a 5-point Likert scale, ranging from 1 (strongly disagree) to 5 (strongly agree).

### Presence in Cinematic Virtual Reality

Following the training program, we observed mean scores greater than or equal to 5.9, out of a maximum score of 7, for all five subscales: *Involvement* (mean 6.22, SD 0.59), *Sensory Fidelity* (mean 5.90, SD 0.81), *Adaptation and Immersion* (mean 6.22, SD 0.61), *Interface Quality* (mean 5.92, SD 1.31), and *Presence* (mean 6.28, SD 0.70). The high subscale scores demonstrate favorable perceptions of the technology and strength of presence in the cine-VR simulations. *Presence* in subscale scores did not differ based on age, gender, race, provider status, or percent of Medicaid patients.

Posttraining, change scores in cultural self-efficacy and diabetes attitudes were correlated with the mean subscale scores of presence. We observed only one significant correlation between the change score in *Affective* self-efficacy and the *Interface Quality* subscale score (*r*=.285, *P*=.03). No other significant correlations were observed between presence in cine-VR subscales and cultural self-efficacy subscale scores or diabetes attitude subscale scores (see Multimedia Appendix 1). These findings did not support the hypotheses that stated that increased presence in cine-VR would be associated with positive changes in cultural self-efficacy subscales and diabetes attitude subscales.

## Discussion

### Principal Findings

In this pilot study, we assessed health care providers’ and administrators’ cultural self-efficacy and diabetes attitudes before and after a 360-degree cine-VR training program. Following the training program, we observed statistically significant improvements in all three cultural self-efficacy subscales: (1) Cognitive, (2) Practical, and (3) Affective. The largest magnitude of effect was observed with the *Practical* subscale, which corresponds to confidence in interviewing patients about social determinants of health. In addition, all five diabetes attitude subscales improved significantly posttraining: (1) *Need for special training*, (2) *Seriousness of type 2 diabetes,* (3) *Value of tight glucose control,* (4) *Psychosocial impact of diabetes,* and (5) *Attitude toward patient autonomy,* with the largest magnitude of change observed in *Psychosocial impact of diabetes*. Lastly, we observed high scores for presence in cine-VR, indicating favorable perceptions of the technology and immersion in the 360-degree virtual environment. Contrary to expectations, only one positive change score in *Affective* self-efficacy was correlated with increased presence in cine-VR.

### Comparison With Prior Work

Effective cine-VR simulations provide a platform to practice and acquire skills that will later translate to clinical outcomes concerning patient care; in addition, they afford participants the opportunity to practice clinical judgment and apply problem-solving skills in a risk-free, replicable clinical environment [29,30]. Cine-VR technology offers new opportunities for clinical assessment and intervention. Advances in virtual reality technologies can now support the creation of low-cost, yet sophisticated, immersive simulations, capable of running on consumer-level computing devices [31]. Compared to traditional video training, the immersive qualities of cine-VR affect the participant’s ability to more strongly retrieve the experience from memory, suggesting that cine-VR experiences become part of an autobiographical associative network, whereas a conventional video experience remains an isolated episodic event [32].

Existing research in narrative health promotion demonstrates the power of culturally tailored stories as engaging content to positively affect attitudes, beliefs, and behaviors. Qualitative results show that the digital storytelling more positively affects participants than traditional face-to-face training on its own, specifically in four growth areas: truth-telling, sense-making, social support, and feeling valued [33]. Research concerning digital storytelling and its uses within health care are only in their infancy in terms of discovering applications and uses. However, recent studies demonstrate that digital stories allow for a deeper understanding of an experience rather than simply hearing an explanation of that experience [34]. Our research supports this finding. Our findings suggest that this innovative cine-VR program can be used to educate providers about type 2 diabetes, social determinants of health, and Appalachian culture, which, in turn, may enhance the delivery of high-quality, evidence-based diabetes care in rural Appalachian Ohio. Additional research is needed to determine the impact of the training on patient care and health outcomes.

Finally, presence describes the extent to which a participant feels present or immersed in a virtual environment [35,36] and is commonly regarded as a necessary mediator that allows real emotions to be activated [37,38]. We hypothesized that higher levels of presence would be associated with positive changes in cultural self-efficacy and diabetes attitudes. We observed only one significant correlation between the change score in *Affective* self-efficacy and the *Interface Quality* subscale score. This finding suggests that participants who felt less distracted by the headset or experienced fewer delays with the simulations showed a greater improvement in the *Affective* self-efficacy scores posttraining. We observed no other significant correlations between positive change scores and presence. This may be explained by the limited variability in presence subscale scores and the overall high level of presence measured in the study. The strength of this 360-degree cine-VR simulation training program is the realism afforded by providing the participant access to the whole environment as compared to traditional virtual reality (eg, animated environments and characters), which has been criticized as being too unrealistic [39].

### Limitations

Limitations of this study include the small homogeneous sample, selection bias, social desirability bias, and lack of a control group. While a final sample of 69 participants is small, our a priori power analysis determined that a sample size of 34 paired participants was sufficient to achieve 80% power and a level of significance of *P*<.05. We successfully doubled the required sample size estimate. However, data from 69 providers and administrators from one geographic region limits the generalizability of the findings to other providers. Further, the predominantly White study sample limits the generalizability to all providers; however, the racial and ethnic distribution of the study sample (83% White) is reflective of the racial and ethnic distribution in southeastern Ohio (95% White) [40]. Next, our findings may be susceptible to selection bias, as individuals who volunteered to participate may have been more willing or motivated to participate in a novel educational program about diabetes, social determinants of health, and Appalachian culture. In addition, the responses may be susceptible to selection bias given the participants may have felt undue pressure to provide positive feedback on the training session. A similar susceptibility to selection bias may be prescribed to the use of new technology encouraging people to provide positive feedback. Finally, this study presents findings from a 3-hour cine-VR training program on type 2 diabetes in rural Appalachia. We did not include a control condition as a comparison group. Future research should use a randomized controlled design to assess the impact of two different educational interventions on providers’ and administrators’ cultural self-efficacy and diabetes attitudes.

### Conclusions

Continuing medical education is an important component of clinical care for all providers. Health care providers and administrators need ongoing and repeated training to help them improve and maintain their knowledge, stay current with the latest developments, address real-world challenges, and learn effective team management skills. Our findings support the notion that 360-degree cine-VR education is an innovative approach to improve cultural self-efficacy and diabetes attitudes among health care providers and administrators. The long-term impact of cine-VR education on cultural self-efficacy and diabetes attitudes needs to be determined.

## Appendix

Multimedia Appendix 1 Correlations among subscale scores of presence in virtual reality, change scores in cultural self-efficacy, and Diabetes Attitude Scale-3 (DAS-3) subscales (n=65).

## Abbreviations

cine-VR: virtual reality cinema
DAS-3: Diabetes Attitude Scale-3
TSET-MHP: Transcultural Self-Efficacy Tool–Multidisciplinary Healthcare Provider
