# Supplementary material for: Using Virtual Reality to Improve Health Care Providers’ Cultural Self-Efficacy and Diabetes Attitudes: Pilot Questionnaire Study
Source: JMIR Diabetes. 2021 Jan 27;6(1):e23708. doi: 10.2196/23708 (PMC7875691; doi:10.2196/23708)
Supplement: Multimedia Appendix 1 [file diabetes_v6i1e23708_app1.docx]

Multimedia Appendix 1: Correlations among subscale scores of presence in virtual reality and change scores in cultural self-efficacy and Diabetes Attitude Scale (DAS-3) subscales (n=65).

| Scale |  | Involvement | Sensory Fidelity | Adaptation & Immersion | Interface Quality | Presence |
| --- | --- | --- | --- | --- | --- | --- |
| TSET |  |  |  |  |  |  |
|  | Cognitive | .152 | .024 | .043 | -.081 | -.065 |
|  | Practical | .088 | .005 | .033 | .037 | .143 |
|  | Affective | .185 | .135 | .214 | .285 | .216 |
| DAS-3 |  |  |  |  |  |  |
|  | Need for special  training | -.062 | .139 | -.022 | -.064 | -.051 |
|  | Seriousness of type 2 diabetes | -.111 | -.072 | -.164 | -.017 | -.162 |
|  | Value of tight glucose control | .037 | .233 | -.014 | -.092 | -.077 |
|  | Psychosocial impact of diabetes | -.018 | .125 | .000 | -.057 | -.023 |
|  | Attitude toward patient autonomy | -.067 | .016 | -.153 | -.003 | -.121 |
